# A Novel Gait Phase Recognition Method Based on DPF-LSTM-CNN Using Wearable Inertial Sensors

**DOI:** 10.3390/s23135905

**Published:** 2023-06-26

**Authors:** Kun Liu, Yong Liu, Shuo Ji, Chi Gao, Shizhong Zhang, Jun Fu

**Affiliations:** School of Mechanical and Aerospace Engineering, Jilin University, Changchun 130025, Chinagaochi21@mails.jlu.edu.cn (C.G.);

**Keywords:** gait phase recognition, wearable inertial sensors, LSTM-CNN, data pre-filtering

## Abstract

Gait phase recognition is of great importance in the development of rehabilitation devices. The advantages of Long Short-Term Memory (LSTM) and Convolutional Neural Network (CNN) are combined (LSTM-CNN) in this paper, then a gait phase recognition method based on LSTM-CNN neural network model is proposed. In the LSTM-CNN model, the LSTM layer is used to process temporal sequences and the CNN layer is used to extract features A wireless sensor system including six inertial measurement units (IMU) fixed on the six positions of the lower limbs was developed. The difference in the gait recognition performance of the LSTM-CNN model was estimated using different groups of input data collected by seven different IMU grouping methods. Four phases in a complete gait were considered in this paper including the supporting phase with the right hill strike (SU-RHS), left leg swimming phase (SW-L), the supporting phase with the left hill strike (SU-LHS), and right leg swimming phase (SW-R). The results show that the best performance of the model in gait recognition appeared based on the group of data from all the six IMUs, with the recognition precision and macro-F1 unto 95.03% and 95.29%, respectively. At the same time, the best phase recognition accuracy for SU-RHS and SW-R appeared and up to 96.49% and 95.64%, respectively. The results also showed the best phase recognition accuracy (97.22%) for SW-L was acquired based on the group of data from four IMUs located at the left and right thighs and shanks. Comparably, the best phase recognition accuracy (97.86%) for SU-LHS was acquired based on the group of data from four IMUs located at left and right shanks and feet. Ulteriorly, a novel gait recognition method based on Data Pre-Filtering Long Short-Term Memory and Convolutional Neural Network (DPF-LSTM-CNN) model was proposed and its performance for gait phase recognition was evaluated. The experiment results showed that the recognition accuracy reached 97.21%, which was the highest compared to Deep convolutional neural networks (DCNN) and CNN-LSTM.

## 1. Introduction

In recent years, the number of stroke patients with hemiplegia has increased rapidly due to the aging of the population. Such patients are unable to live independently due to the impairment of lower limb functions, which has brought a heavy burden to families and society [1]. Intelligent lower limb rehabilitation equipment can provide effective rehabilitation training and motion assistance for patients [2,3]. Gait recognition, as one of the key technologies in the control part of rehabilitation equipment, plays an important role in the motion assistance process [4,5,6]. Gait, or walking, is a cyclic movement exhibiting reoccurring patterns while maintaining static and dynamic balance. A complete gait cycle can be divided into the supporting phase and swinging phase. Both feet contact the ground in the supporting phase consuming about 60% of the whole gait cycle and only one foot contacts the ground in the swinging phase consuming about 40% of the whole gait cycle [7]. The gait recognition results with high accuracy can not only improve the control effect of the rehabilitation equipment developed based on the human–computer cooperative control strategy but also be applied to the clinical treatment plan formulation of stroke or other lower limb dysfunction diseases [8].

The threshold method was generally used to complete gait recognition in most previous studies [9], but the threshold value is an empirical value which could be affected by the subjective factors of the designer. With the rapid development of artificial intelligence technology, various types of neural network models have been applied to gait recognition such as the image-based method and wearable inertial sensor-based method. The image-based gait recognition method generally obtains a series of human motion images from a high-speed camera system and then uses the neural network model to extract the feature information for gait recognition [10,11,12]. Although the accuracy of the image-based gait recognition method is high, it is difficult to apply this method to real-time control of rehabilitation equipment due to the high cost and inconvenient installation. The application of inertial sensors in gait recognition has attracted more and more researchers’ attention due to its advantages of small size, low cost, and high accuracy [13,14]. Based on the kinematic parameters of lower limb joints collected by inertial sensors, the neural network model for gait recognition with high accuracy can be realized. An integrated network model SBLSTM was proposed and recognized the gait phases effectively [15]. A Deep Convolutional Neural Networks (DCNN) recognition method based on IMU was published, the method performed best in the recognition of the swing phase and worst in the recognition of the terminal stance [16]. A new multi-model LSTM network for gait recognition was proposed in [17], the model performed better than other LSTM models. A graph convolutional network model (GCNM) for gait phase classification to control a lower limb exoskeleton system was presented [18], the model could recognize four gait phases of one leg with high accuracy. A novel gait pattern recognition method combined with LSTM and CNN was proposed and the recognition accuracy unto 97.78% [19]. However, the purpose of all the aforementioned methods mainly focused on improving the accuracy of gait recognition without attention to the numbers and locations of used sensors. The accuracy of gait recognition using neural network models with two IMUs attached to thighs, shanks, and feet, respectively was compared [20]; however, only two IMUs were used.

The LSTM model is good at processing time-series data, and the CNN is an expert in processing data with spatial structure characteristics. Therefore, this paper combined the advantages of the two models, and a gait recognition method based on LSTM-CNN neural network model was proposed. The self-developed wireless sensor system including the wearable inertial sensor sub-system and force platform sub-system could simultaneously capture six joint angles and ground reaction force, then the collected data could be used to make training and verification data sets. The difference in the gait recognition performance of the LSTM-CNN model was estimated using different groups of input data collected by seven different IMU grouping methods. Based on the results of the first part of the experiment, this paper proposes a novel gait recognition method based on DPF-LSTM-CNN and verifies its performance in the second part of the experiment. Ulteriorly, a novel gait recognition method based on Data Pre-Filtering Long Short-Term Memory and Convolutional Neural Network (DPF-LSTM-CNN) model was proposed and its performance for gait phase recognition was evaluated.

## 2. Methods

### 2.1. Data Collection

**Figure 1:**
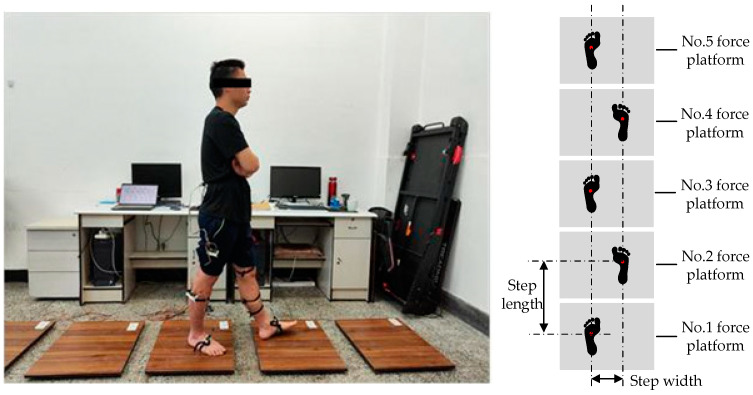
The actual application scenario of the sensor system.

In order to meet the data set requirements for neural network model training in this paper, our team has developed a wireless sensor system including a wearable inertial sensor sub-system (IMU) and force platform sub-system. The system could simultaneously collect the angle signals of six joints of lower limbs and real-time plantar force signals during human gait. JY901 was selected as the inertial sensor to collect joint angles. The force platform sub-system consisted of six independent force measuring plates to measure the foot force when the human is walking. The Arduino Nano (Shenzhen Mingjiatai Electronics Co., Ltd., Shenzhen, China) was selected as the master control unit and collected joint angles and plantar force signals at a frequency of 100 Hz during human walking, and simultaneously transmits real-time data with the host computer. The nrf24l01 (Shenzhen Mingjiatai Electronics Co., Ltd.) wireless sensor was selected to realize wireless data transmission of the sensor system, as shown in Figure 1.

### 2.2. Data Preprocessing

In order to make the collected data have the same dimensions and improve the recognition accuracy of the neural network model, it is necessary to preprocess the original data collected by the IMUs. Linear interpolation and data normalization are common methods for data preprocessing include. Linear interpolation can solve the packet loss problem of sensor data during transmission, while data normalization can limit the collected data with different amplitudes and dimensions to the specified range through certain mathematical operations to obtain standardized dimensionless data. After the above data processing, the training complexity of the neural network can be reduced and the accuracy of gait recognition can be improved. In this paper, the raw data was divided with different gait phase information using the sliding window segmentation method and each sliding window contains 20 samples.

The mathematical expression of linear interpolation is as follows:

(1) $$y=\frac{x-x_{0}}{x_{1}-x_{0}}(y_{1}-y_{0})+y_{0},$$

where (*x*_0_, *y*_0_) and (*x*_1_, *y*_1_) represent the known sampling point information, and (*x*, *y*) is the unknown sampling point information at the *x* sampling point.

The mathematical expression of data normalization is as follows:

(2) $${\tilde{x}}_{i}=\frac{x_{i}-x_{\min}}{x_{\max}-x_{\min}}(y_{\max}-y_{\min})+y_{\min},$$

where *x_i_* is the element *i* in the input vector before being normalized, and *x_max_* and *x_min_* are the maximum and minimum values of *x_i_*, respectively. *y_max_* and *y_min_* are the upper and lower limits of the normalized data, the values are set to 1 and −1, respectively.

The whole gait process was divided into the following four different gait phases in this paper: supporting phase with right hill strike (SU-RHS), left leg swing phase (SW-L), supporting phase with left hill strike (SU-LHS), and right leg swing phase (SW-R). Since the category of gait phase is discrete feature, this paper used one hot vector to represent the real distribution for the output of gait phase recognition model, all gait sub-phases are denoted as follows:

(3) $$\left\{ \begin{array}{cc} \mathrm{SU}-\mathrm{RHS} & \;[1\;0\;0\;0]\\ \mathrm{SW}-L & \;[0\;1\;0\;0]\\ \mathrm{SU}-\mathrm{LHS} & \;[0\;0\;1\;0]\\ \mathrm{SW}-R & \;[0\;0\;0\;1] \end{array}.\right.$$

In order to record and calculate the label of IMUs training data, the plantar force data measured by the self-made force platform system was used as the reference standard [21,22]. The research work in [23] showed that when the threshold value of foot force was selected as 20 N, the contact between the foot and the force platform could be accurately judged. When the measured foot force was less than 20 N, the foot was considered to have completely disengaged from the force platform; when the measured foot force was greater than 20 N, the foot was considered to have completely contacted with the force platform.

### 2.3. Structure of LSTM-CNN Neural Network

#### 2.3.1. Structure of LSTM

**Figure 2:**
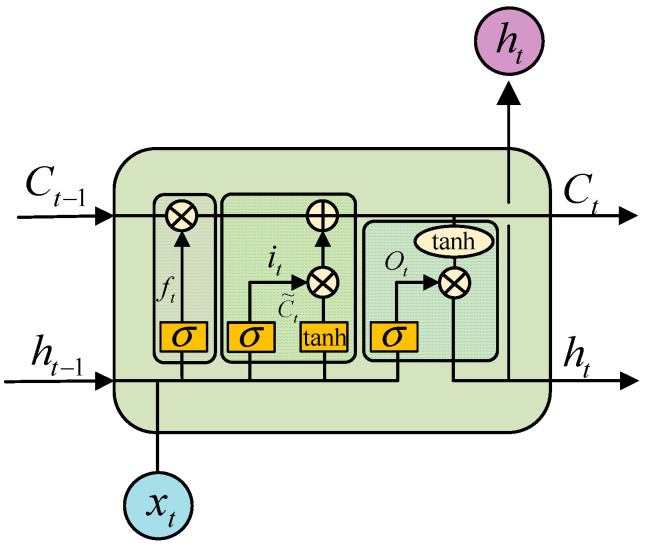
Internal structure of LSTM unit.

As an improved neural network model based on recurrent neural network (RNN), LSTM neural network is mainly used to solve the problem of gradient disappearance and gradient explosion during long sequence training. In short, LSTM can perform better in longer time series than ordinary RNN. The reason why LSTM can solve the long-term dependency problem of RNN is that it introduces a gate mechanism to control the flow and loss of features. The storage unit of LSTM contains three gate structures: forgetting gate, input gate, and output gate. The forgetting gate determines the information that needs to be retained and discarded from the previous storage unit, the input gate parses the information that needs to be updated to the storage unit, and the output gate determines the output information according to the input and storage unit [24,25]. It is precise because LSTM has a unique internal structure that it can process time series signals well. The internal structure of LSTM unit is shown in Figure 2.

(4) $$\left\{ \begin{array}{l} i_{t}=\sigma \left(W_{i}h_{t-1}+W_{i}X_{t}+b_{i}\right)\\ f_{t}=\sigma \left(W_{f}h_{t-1}+W_{f}X_{t}+b_{f}\right)\\ O_{t}=\sigma \left(W_{O}h_{t-1}+W_{O}X_{t}+b_{O}\right) \end{array}.\right.$$

*C_t_* Update formula and output of the whole unit *h_t_* are as follows:

(5) $$\left\{ \begin{array}{l} h_{t}=O_{t}\times \tanh(C_{t})\\ C_{t}=f_{t}\times C_{t-1}+i_{t}\times \tanh\left(W_{c}h_{t-1}+W_{c}X_{t}+b_{c}\right) \end{array},\right.$$

where *x_t_* represents input, *h_t_* represents the hidden layer state at time *t*, and [*W*] and [*b*] represent the weight matrix and deviation term of LSTM unit, respectively.

#### 2.3.2. Structure of CNN

**Figure 3:**
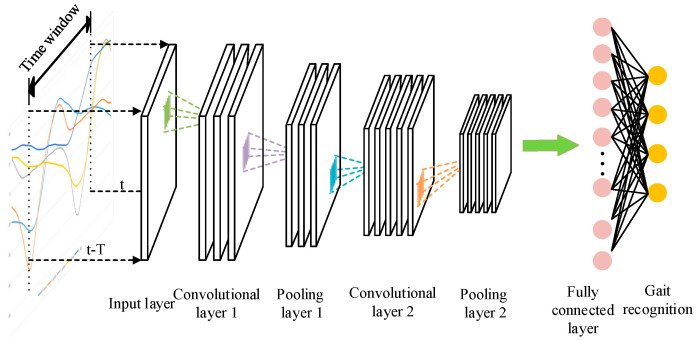
Structure of CNN.

The structure of CNN neural network model is shown in Figure 3. CNN network mainly includes three parts: convolution layer, pooling layer, and full connection layer. Among them, the convolution layer is mainly used to extract the characteristics of the input data, the pooling layer is mainly used to compress the amount of data and parameters, reduce over fitting, and the full connection layer is used for classification. As the most important part of the CNN network, the convolution layer mainly uses the convolution kernel to extract features. The output of the convolution layer can be expressed by the following formula:

(6) $$x_{j}^{l}=f\left(\sum_{i=1}^{M}\left(x_{i}^{l-1}\times W_{j}^{l}\right)+b_{j}^{l}\right),$$

where *x_j_^l^* is the output feature of the *l*-layer, *W_j_^l^* is the convolution kernel weight of the *l*-layer, *x_i_^l^****^−^***^1^ is the input data feature of the *l*-layer, *b_j_^l^* is the corresponding offset, and *f* is the activation function. ReLU is used as activation function in this paper.

Pooling layer is connected behind convolution layer to reduce over fitting during network training. There are mainly two pooling methods: maximum pooling and average pooling. When the maximum pooling method is adopted, the point with the largest eigenvalue in the pooled area is taken as the new feature point. When the average pooling method is adopted, the average eigenvalue in the pooled area is taken as the new feature point. In this paper, the maximum pooling method is adopted. The input data is transformed into the final feature samples after being processed by multi-layer convolution layer and pooling layer, and the full connection layer maps these features to the sample label space to enable the CNN network to realize gait recognition.

#### 2.3.3. Structure of LSTM-CNN

**Figure 4:**
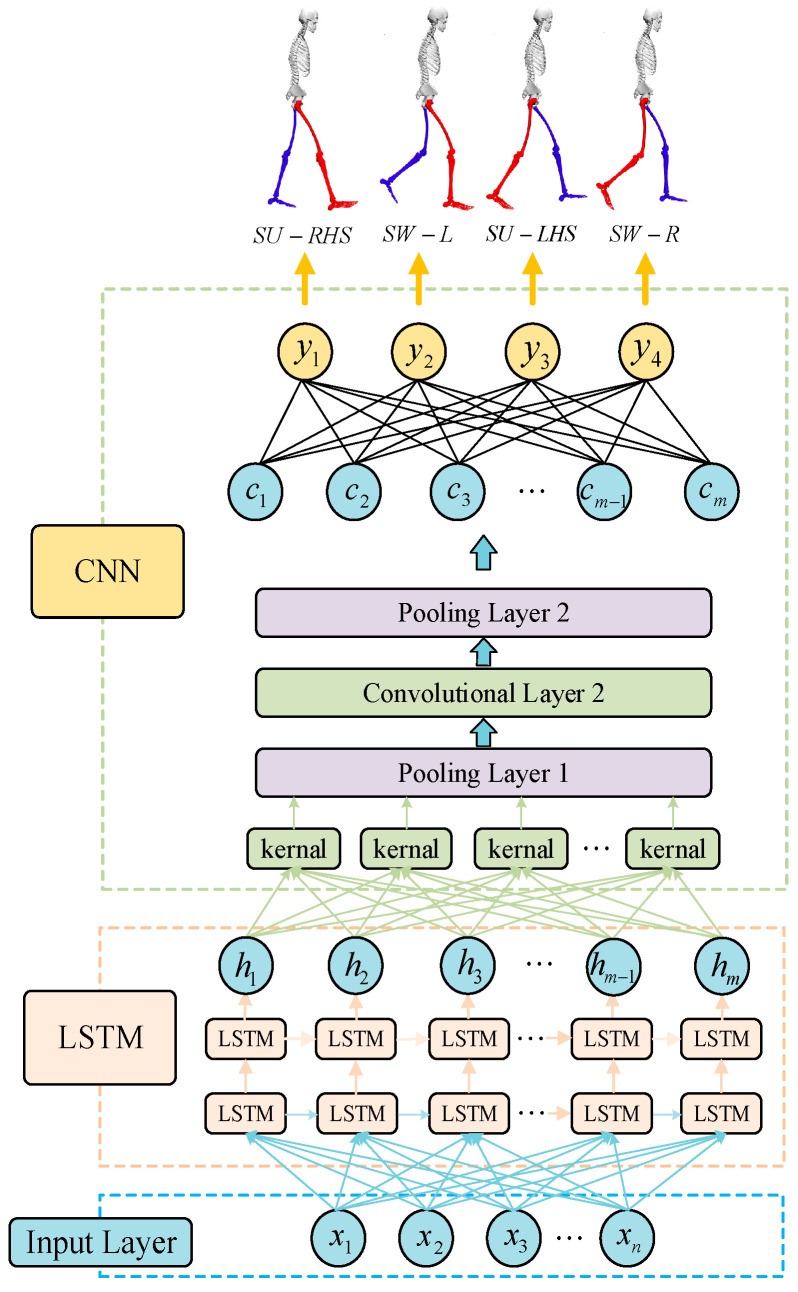
Structure of LSTM-CNN.

Figure 4 shows the LSTM-CNN neural network model structure used for gait recognition in this paper including a double-layer LSTM network and a double-layer CNN network. In LSTM, the two hidden layers contain 128 units, and the activation function used by the hidden layer is the tanh function. In CNN, the two convolution layers have 64 and 128 convolution kernels, respectively, the size of the convolution kernels is 1 × 3, and the convolution step is 1. The size of the pool group in the two layers of pooling layer is 2 × 2, the step size of the first layer of pooling layer is 2, and the step size of the second layer of pooling layer is 1. The size of the pool group in the two layers of pooling layer is 2 × 2, the step size of the first layer of pooling layer is 2, and the step size of the second layer of pooling layer is 1. In this paper, cross entropy is used as the loss function of neural network model, and Adam optimizer algorithm is used to optimize the parameters of LSTM-CNN network.

### 2.4. Evaluation Method

In order to evaluate the performance of the neural network model used in this paper in gait recognition, accuracy (Acc), and F1-score are used as performance evaluation indicators.

The formula of Acc is as follows:

(7) $$\mathrm{Acc}=\frac{\mathrm{TP}+\mathrm{TN}}{\mathrm{TP}+\mathrm{TN}+\mathrm{FP}+\mathrm{FN}},$$

where TP, TN, FP, and FN represent true positive, true negative, false positive, and false negative, respectively.

The F1-score is calculated based on two evaluation indicators: precision (Pre) and recall (Rec). Due to the interaction between precision and recall, it is sometimes difficult to compare the performance differences between different methods through these two indicators. The F1-score combines the results of precision and recall, which can represent the performance of the model in terms of precision and recall. When the F1-score is high, both accuracy and recall are high. However, F1-score, precision, and recall are generally used for the evaluation of binary tasks, which is not applicable to gait recognition, a typical multi classification task studied in this paper. Macro-F1, macro-precision, and macro-recall proposed in [26] are usually used to evaluate the model performance of multi category tasks. The macro-precision, macro-recall, and macro-F1 score are calculated as follows:

(8) $$\left\{ \begin{array}{l} \mathrm{Pr}e_{macro}=\frac{1}{n}\sum_{i=1}^{n}\left(\frac{\mathrm{TP}}{\mathrm{TP}+\mathrm{FP}}\times 100\%\right)\\ \mathrm{Rc}e_{macro}=\frac{1}{n}\sum_{i=1}^{n}\left(\frac{\mathrm{TP}}{\mathrm{TP}+\mathrm{FN}}\times 100\%\right)\\ F1_{macro}=\frac{2\times \mathrm{Pr}e_{macro}\times \mathrm{Rc}e_{macro}}{\mathrm{Pr}e_{macro}+\mathrm{Rc}e_{macro}}\times 100\% \end{array}.\right.$$

In addition to accuracy and macro-F1 score, confusion matrix is often used to evaluate the performance of neural network models [27]. The mathematical expression of the confusion matrix is as follows:

(9) $$C=\left(\begin{array}{llll} c_{11} & c_{12} & c_{13} & c_{14}\\ c_{21} & c_{22} & c_{23} & c_{24}\\ c_{31} & c_{32} & c_{33} & c_{34}\\ c_{41} & c_{42} & c_{43} & c_{44} \end{array}\right)$$

The formula of each element in the matrix is as follows:

(10) $$c_{ij}=\frac{n_{ij}}{n_{i}},$$

where *n_ij_* represents the amount of data that gait phase *i* is recognized as *j*; *n_i_* is the total number of testing data in gait phase *i*.

## 3. Experiment and Results of Gait Recognition

**Figure 5:**
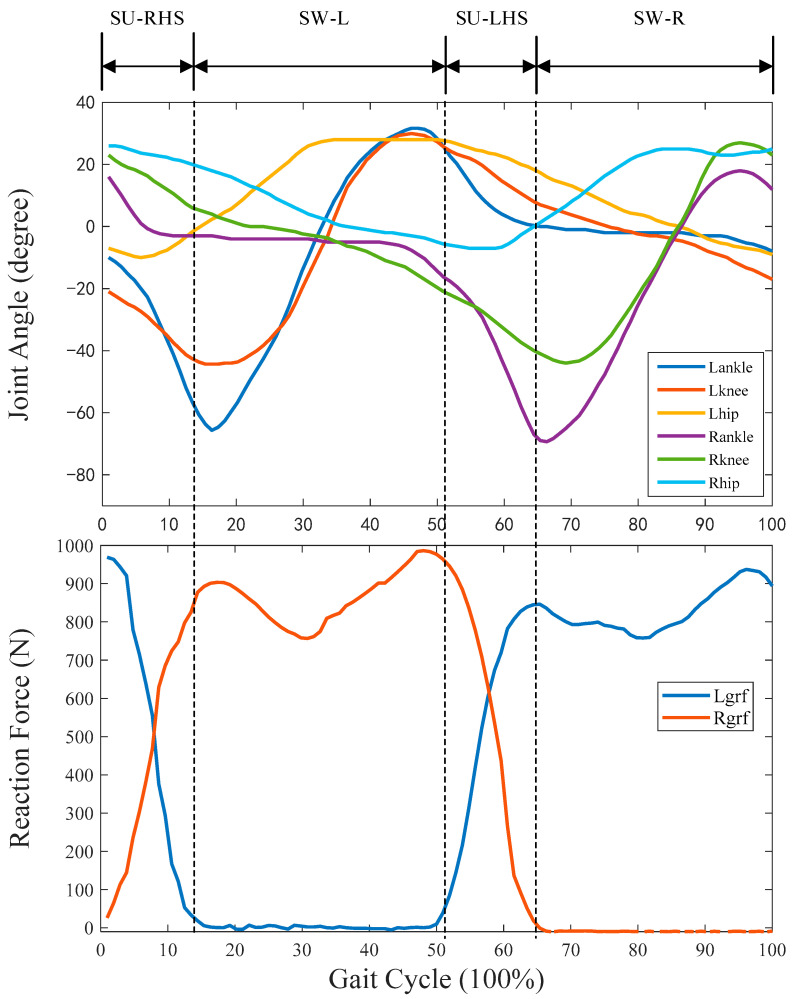
Gait phase division diagram. The Lgrf represents the left ground reaction force and Rgrf represents the right ground reaction force.

Ten healthy young adults (age = 26 ± 3 years, mass = 70 ± 7.3 kg, height = 175 ± 5.5 cm) and ten healthy elderly adults (age = 64.5 ± 7.6 years, mass = 61.6 ± 10.8 kg, height = 167.3 ± 8.4 cm) without known lower limb musculoskeletal or neurological dysfunction participated in this study. Written and verbal instructions of testing procedures were provided, and written consent was obtained from each subject prior to the experiment. The experimental protocol was approved by the Human Ethical Review Committee of Jilin University (No. 2023-233). After getting familiar with the experimental process, the subjects wore the adjusted sensors to complete walking. In the experiment, walking data were collected at slow speed (about 1.2 m/s), medium speed (about 1.5 m/s), and fast speed (about 1.8 m/s) speeds, respectively. Due to the limited length of the self-developed force platform, each subject needs to complete fifteen experiments. So, for each subject, the experiment collected walking data of 30 gait cycles (10 gait cycles for each walking speed × three walking speeds), and the experiment collected walking data of 600 gait cycles (30 gait cycles for each subject × 20 subjects) in total. Takea 500 gait cycle walking data including 25 gait cycle walking data for each subject for LSTM-CNN model training, and the remaining 100 gait cycle walking data for subsequent validation experiments. The walking data of subject 1 in one gait cycle are shown in Figure 5. In this paper, Matlab2021a was used as the training software for the neural network model. The hardware configuration includes i9-11900k, GTX2070ti graphics card, and 32 GB memory.

### 3.1. The Training of LSTM-CNN

**Figure 6:**
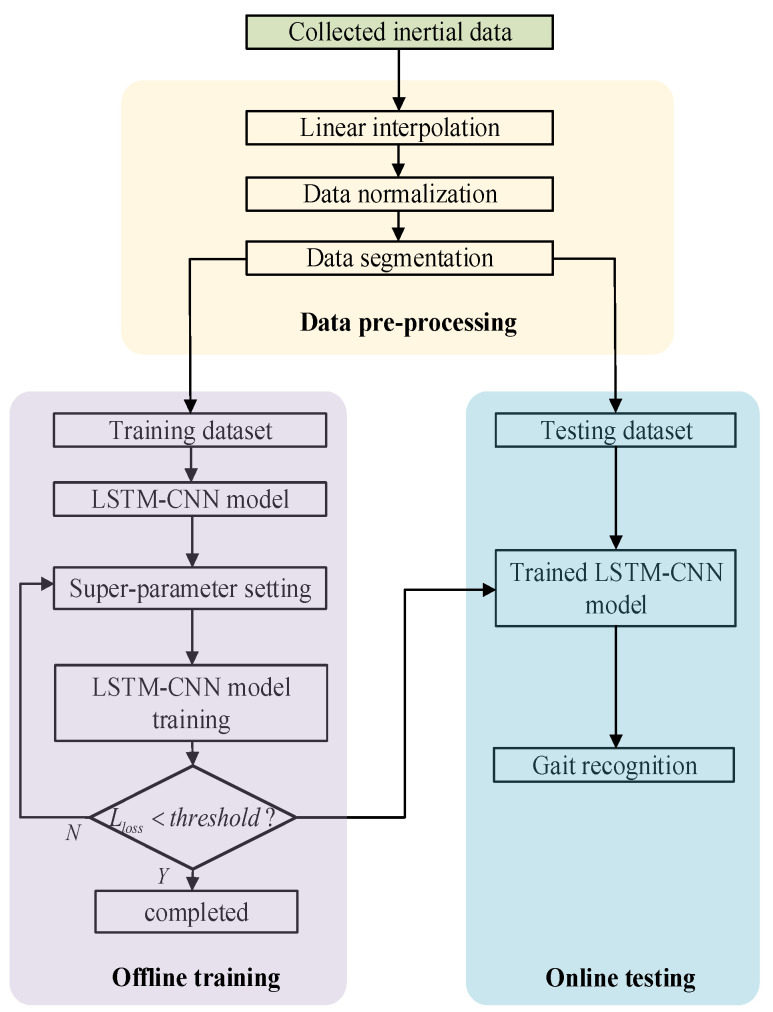
Gait pattern recognition framework based on LSTM-CNN.

The joint angles collected by IMUs were processed according to the above data preprocessing method. The sensor system used in this paper can collect six joint angles of human lower limbs. When the data collected by sensors with different position combinations was used as the input of the neural network model, the model may have different performance in gait recognition. In this paper, the experiments were divided into seven groups according to different sensor position combinations, as shown in Table 1. The gait recognition flow chart based on LSTM-CNN is shown in Figure 6, in which the training dataset accounts for 70% of the total dataset, and the test dataset accounts for 30% of the total dataset. Through iterative training, when the loss value exceeds the threshold value, the model training is considered complete.

Table 2 shows the experimental results statistics of the 7 groups of experiments on the gait phases of SU-RHS, SW-L, SU-LHS and SW-R, which include precision, recall and F1-score. Among them, LSTM-CNN performed best in the seventh group of experiments. Except that the precision of SU-LHS is 89.08%, all other indexes are greater than 90%. The performance of the model was poor in the third group of experiments, in which the F1-score and recall are lower than 90%. In addition, the F1-score of SU-RHS is the only one that is lower than 80% of all indices, and the F1-score is 78.64%. When the data collected by two IMUs was used as the input of the model, LSTM-CNN performed poorly, and the average F1-score in groups 1 to group 3 is 85.25%. When the data collected by four IMUs were used as the input of the model, the performance of LSTM-CNN was significantly improved, and the average F1-score in group 4 to group 6 is 91.80%. The model performed best in the seventh group of experiments, with an average F1-score of 95.61%.

**Figure 7:**
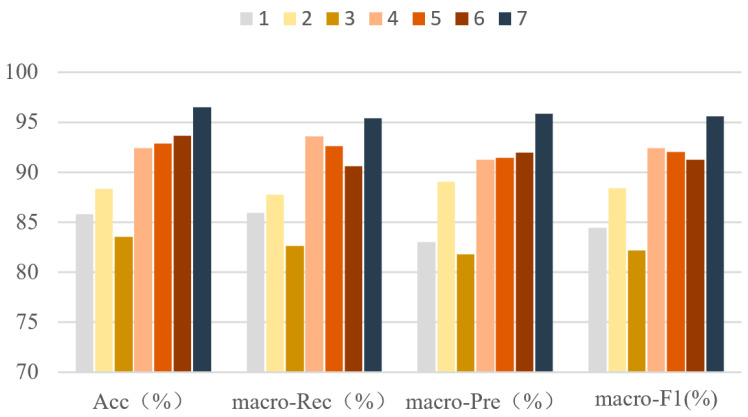
Accuracy, macro-recall, macro-precision, and macro-F1 of LSTM-CNN in 7 experiments.

**Figure 8:**
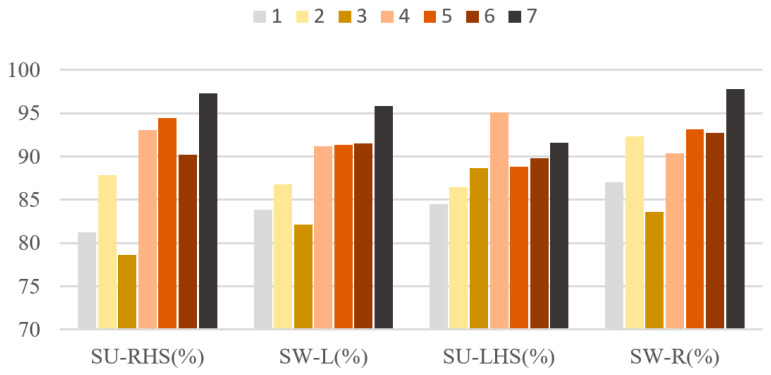
F1-scores of LSTM-CNN for four gait phases recognition in 7 experiments.

**Figure 9:**
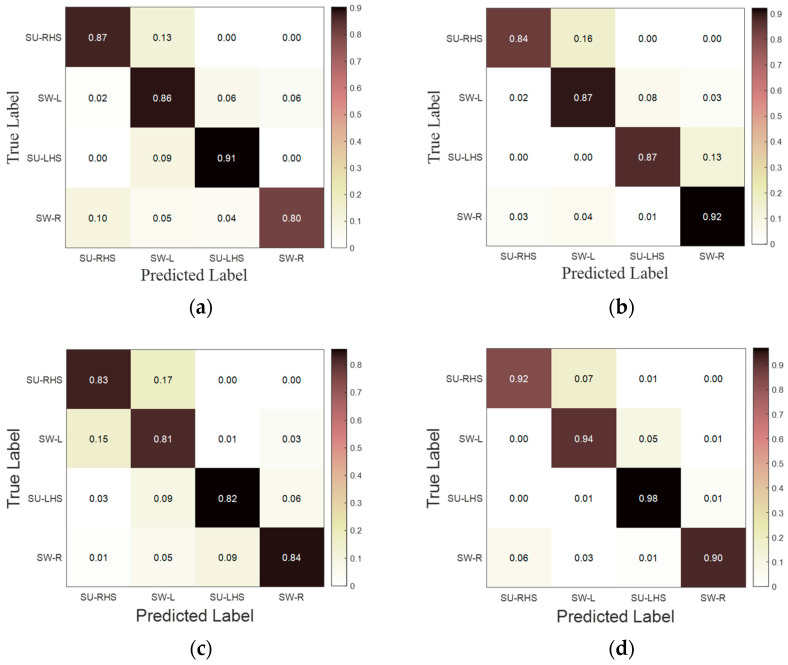

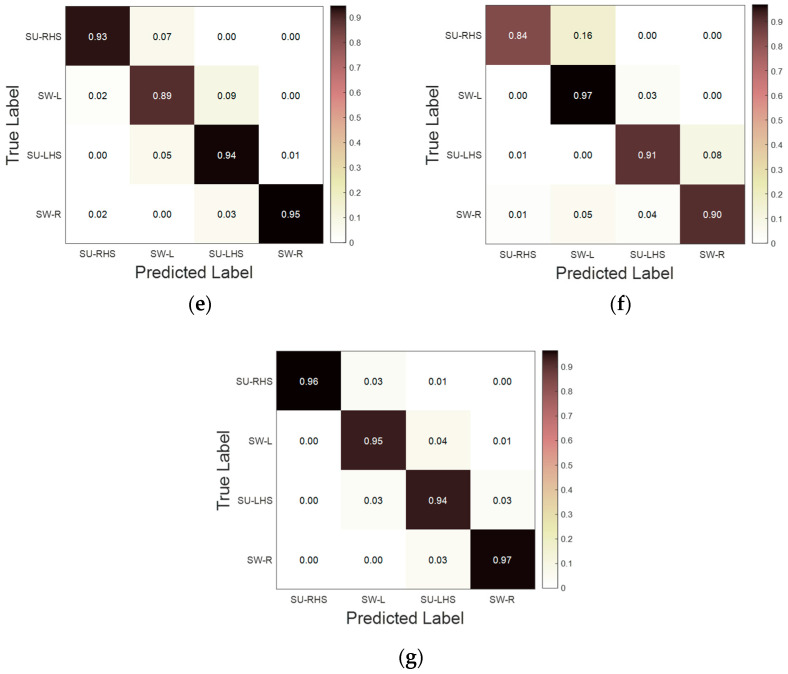
Confusion matrix of LSTM-CNN in 7 experiments. (**a**) Group 1; (**b**) Group 2; (**c**) Group 3; (**d**) Group 4; (**e**) Group 5; (**f**) Group 6; (**g**) Group 7.

Figure 7 shows the experimental statistical results of accuracy, macro-precision, macro-recall, and macro-F1 using the LSTM-CNN neural network model in seven groups of experiments. It can be seen from Figure 7 that the accuracy, macro-precision, macro-recall, and macro-F1 in the third group of experiments are 83.56%, 81.81%, 82.61%, and 82.21%, respectively, and 96.49%, 95.84%, 95.45% and 95.64%, respectively in the seventh group of experiments. The indexes are the smallest and largest, respectively in the third group and seventh group. In addition, each index in group 1 to group 3 is less than 90%. Figure 8 shows the F1-score of LSTM-CNN for SU-RHS, SW-L, SU-LHS and SW-R sub-phases, it can be seen from Figure 8 that the F1-score in the SU-LHS is the highest in the fourth group of experiments which is 95.11%, and the maximum F1-scores in the other three phases are in the seventh group of experiments which is 97.26%, 95.84% and 97.77%, respectively. Figure 9 represents the confusion matrix of seven groups of experiments. It can be seen from Figure 9 that the recognition accuracy of SU-RHS and SW-R is highest in the seventh group of experiments which is 95.26% and 96.66%, respectively, the recognition accuracy of SW-L is highest in the sixth group of experiments which is 97.22%, and the recognition accuracy of SU-LHS is highest in the fourth group of experiments which is 97.86%.

### 3.2. Experiment and Results Based on DPF-LSTM-CNN

**Figure 10:**
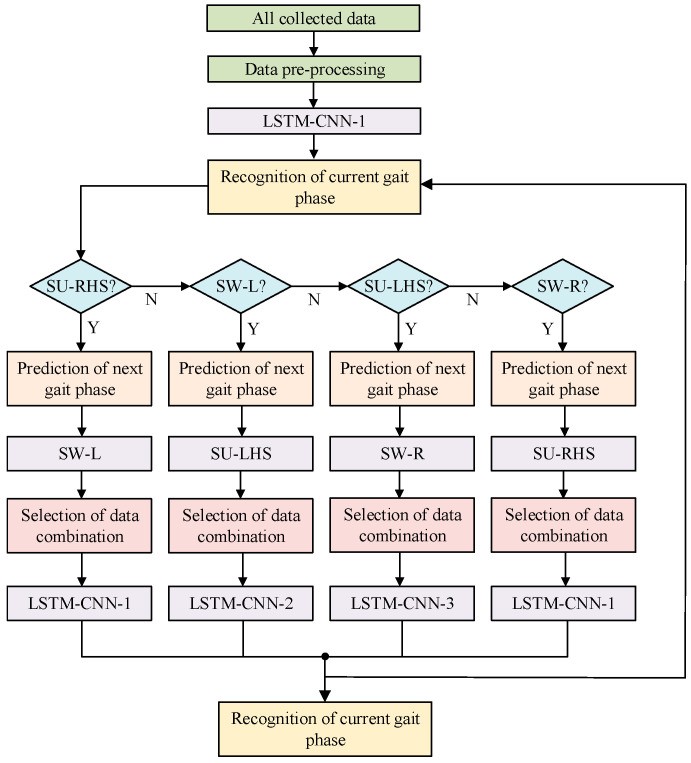
Gait recognition flow chart based on DPF-LSTM-CNN.

Based on the above experimental results, it can be found that the best gait phase recognition performance of LSTM-CNN appeared based on the group of data from all the six IMUs; meanwhile, the recognition accuracies for SU-RHS and SW-R out of the four phases are also the highest. Therefore, the corresponding trained model derived from the group of data from all the six IMUs is defined as LSTM-CNN-1. However, the highest recognition accuracy for SW-L sub-phase appeared based on the group of data from the four IMUs located on the shanks and feet, then the corresponding trained model was defined as LSTM-CNN-2 in this case. Comparatively, the highest recognition accuracies for SU-LHS sub-phase appeared based on the group of data from the four IMUs located on the thighs and shanks, then the corresponding trained model was defined as LSTM-CNN-3 in this case. The four phases (SU-RHS, SW-L, SU-LHS, SW-R) appear in a fixed sequence throughout a complete gait cycle and continue to circulate in a durative gait task. Conclusively, a gait recognition method defined as DPF-LSTM-CNN is innovatively proposed and its specific execution procedure is shown in Figure 10. As the beginning of the recognition procedure, the group of data from all the six IMUs should be input to the trained model LSTM-CNN-1 to recognize the current gait phase, and thus the sequential phase out of the four phases in in a fixed sequence is also obviously predicted. Later, the aforementioned sequential gait phase has converted to the current gait phase whose corresponding trained model (LSTM-CNN-1, 2 or 3) with the highest recognition accuracy could be accurately determined as previously elaborated. Resultantly, the current gait phase was recognized with the highest recognition accuracy. Continuously, repeat the processing procedure in a loop to achieve real-time recognition of gait phases until the walking is over. In order to improve the accuracy of the neural network model in gait recognition and reduce error accumulation, when the gait at five consecutive times is recognized as the same gait phase, the recognition result of the model is considered effective.

In order to verify the performance of this method in gait recognition, 20% of gait data in each subject’s gait data that did not participate in neural network model training were taken as validation data sets, LSTM-CNN-1, and DPF-LSTM-CNN were used for gait recognition to compare the differences between the two methods.

**Figure 11:**
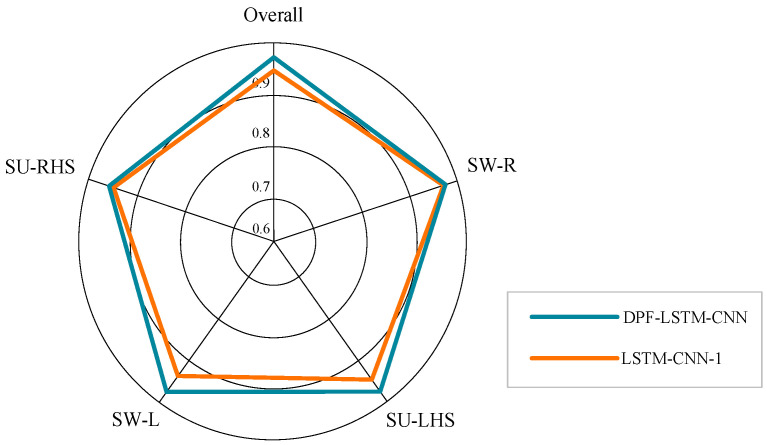
Gait phase recognition accuracy for each phase and overall of subject 1.

Table 3 shows the accuracy and macro-F1 of gait recognition using the two methods for 20 subjects as verification objects, respectively. It can be seen from Table 3 that the average accuracy and average macro-F1 of gait recognition using LSTM-CNN-1 for 20 subjects as verification objects are 94.17% and 94.38%, respectively. The average accuracy and average macro-F1 of gait recognition using DPF-LSTM-CNN were 96.73% and 96.48%, respectively. Figure 11 shows the recognition accuracy of the two methods for four gait phases when subject 1 was the verification object. The accuracy of LSTM-CNN-1 for SU-RHS, SW-L, SU-LHS and SW-R sub-phase is 94.85%, 94.13%, 95.25% and 96.84%, respectively. The accuracy of DPF-LSTM-CNN for SU-RHS, SW-L, SU-LHS, and SW-R sub-phase was 96.83%, 97.73%, 98.47%, and 96.88%, respectively.

To further verify the performance of DPF-LATM-CNN, we compare this method with several algorithms including Deep convolutional neural networks (DCNN) [16], Convolutional, and Long Short-Term Memory neural networks (CNN-LSTM) [13]. Table 4 showed the recognition accuracy and Macro-F1 of the DPF-LSTM-CNN, DCNN, and CNN-LSTM. It can be seen from Table 4 that the accuracy and macro-F1 of the gait recognition based on DPF-LSTM-CNN are the highest which is 97.21% and 96.48%, respectively.

## 4. Discussion

In this paper, LSTM-CNN neural network model was used to recognize four gait phases in the gait process based on self-developed wearable wireless sensor system. The paper has analyzed the performance of the model in gait recognition using different groups of input data collected by seven different IMU grouping methods. It can be seen from Figure 7 that in the experiments of the first to third groups when only the data collected by two IMUs were used as the input of the model, the accuracy, macro-recall, macro-precision, and macro-F1 of LSTM-CNN in recognizing the four gait phases are less than 90%. In the experiments of the fourth to seventh groups, when more than 4 IMUs were used, each index exceeds 90%, and in the experiments of group 7, each index exceeds 95%. The above results show that when the feature dimensions input into LSTM-CNN become higher, the comprehensive performance of the model for gait recognition will be improved accordingly. In this paper, when the data of all six IMUs were used as the input of the model, LSTM-CNN performs best in gait recognition, with accuracy, macro-recall, macro-precision, and macro-F1 of 96.49%, 95.45%, 95.84, and 95.64%, respectively.

Although the comprehensive performance of the LSTM-CNN will be improved when the input feature dimension is higher, LSTM-CNN shows different characteristics when recognizing four different gait phases. It can be seen from Figure 8 that the maximum value of F1-scores of SU-RHS, SW-L, SU-LHS, and SW-R are not all in the seventh group of experiments. When the model was used to recognize SU-LHS sub-phase, the F1-score in group 4 is 97.86%, which is higher than 94.23% in group 7. It can be seen from Figure 9 that LSTM-CNN still performs best in the seventh group of experiments for the recognition accuracy of the four gait phases with an average accuracy of 95.60%. However, there are some differences in the recognition of each gait phase. LSTM-CNN has the highest recognition accuracy in the seventh group of experiments only when recognizing SU-RHS and SW-R sub-phases, with an accuracy of 95.26% and 96.66%, respectively. The highest accuracy is 97.22% in the sixth group of experiments when recognizing SW-L, and 97.86% in the fourth group of experiments when recognizing SU-LHS. The above results show that the performance of the neural network model may be better by using fewer sensors when recognizing a specific gait phase. Applying the above conclusions to the research and development of lower limb rehabilitation equipment, on the basis of improving the accuracy of gait recognition, it can not only reduce the number of sensors required in the equipment, but also reduce the computing pressure of the equipment control system, so that the equipment does not rely on high-performance microprocessors, and effectively reduce the cost of equipment.

It can be seen from Table 3 that when DPF-LSTM-CNN model was used for gait recognition, the accuracy, and macro-F1 have been significantly improved. The average accuracy of the model is improved from 94.17% to 96.73%, and the average macro-F1 is increased from 94.38% to 96.48% when 20 subjects were used as validation objects, respectively. Taking subject 1 as an example, it can be seen from Figure 11 that the recognition accuracy of DPF-LSTM-CNN and LSTM-CNN-1 for SW-R recognition is similar, with 96.48% and 96.88%, respectively. Except for the recognition of SW-R sub-phase, the accuracy of DPF-LSTM-CNN in recognizing the other three gait phases is significantly higher than that of LSTM-CNN-1 model. Furthermore, compared to the DCNN and CNN-LSTM, the DPF-LSTM-CNN performed better in gait recognition.

## 5. Conclusions

Based on the self-developed wearable wireless sensor system, the performance of the LSTM-CNN neural network model in gait recognition was analyzed using different groups of input data collected by seven different IMU grouping methods. The experimental results show that when all six IMU were used, the overall performance of the model in gait recognition is the best, and its accuracy and macro-F1 are 96.49% and 95.64%, respectively. The recognition accuracy of the model for SU-RHS and SW-R is the highest under this condition with 95.62% and 96.66%, respectively. When using the data of IMU located at the left and right thighs and shanks, the model has the highest accuracy for SW-L, with an accuracy of 97.22%. When using the data of IMU located at the left and right shanks and feet, the model has the highest accuracy for SU-LHS, with an accuracy of 97.86%. Based on the above conclusions, this paper has proposed a novel gait recognition method based on DPF-LSTM-CNN and makes an experimental verification of its performance in gait recognition. The experimental results show that the method performed better in gait recognition compared to the DCNN and CNN-LSTM. The model gait recognition method based on DFP-LSTM-CNN proposed in this paper can provide a better reference for accurate control of lower limb motion rehabilitation assistive devices.

## Figures and Tables

**Table 1:** The groups of experiments depend on the IMU position combination.

Groups	Position Combination of IMU
1	Left and right thighs
2	Left and right shanks
3	Left and right feet
4	Left and right thighs and shanks
5	Left and right thighs and feet
6	Left and right shanks and feet
7	Left and right thighs, shanks and feet

**Table 2:** Comparison of precision, recall, and F1-score of different groups.

Groups	Gait Phases	Pre (%)	Rec (%)	F1-Score (%)
1	SU-RHS	76.43	86.67	81.23
	SW-L	81.89	85.84	83.82
	SU-LHS	78.75	91.11	84.48
	SW-R	95.01	80.27	87.02
2	SU-RHS	91.89	84.17	87.86
	SW-L	86.79	86.79	86.79
	SU-LHS	85.86	87.11	86.48
	SW-R	91.78	92.91	92.34
3	SU-RHS	74.43	83.35	78.64
	SW-L	83.06	81.22	82.13
	SU-LHS	86.50	81.95	88.63
	SW-R	83.26	83.92	83.59
4	SU-RHS	93.60	92.46	93.03
	SW-L	88.39	94.15	91.18
	SU-LHS	92.51	97.86	95.11
	SW-R	90.65	90.03	90.34
5	SU-RHS	96.17	92.77	94.44
	SW-L	94.08	88.79	91.36
	SU-LHS	84.00	94.24	88.83
	SW-R	91.63	94.72	93.15
6	SU-RHS	97.28	84.13	90.23
	SW-L	86.38	97.22	91.48
	SU-LHS	88.33	91.22	89.75
	SW-R	95.88	89.78	92.73
7	SU-RHS	98.96	95.62	97.26
	SW-L	96.42	95.27	95.84
	SU-LHS	89.08	94.23	91.58
	SW-R	98.91	96.66	97.77

**Table 3:** Accuracy and macro-F1 of gait recognition using LSTM-CNN-1 and DPF-LSTM-CNN for 20 subjects as verification objects, respectively.

Subject	LSTM-CNN-1		DPF-LSTM-CNN	
	Acc (%)	Macro-F1 (%)	Acc (%)	Macro-F1 (%)
1	94.84	93.67	97.63	97.04
2	96.37	96.11	97.84	97.29
3	94.12	95.23	95.29	96.24
4	92.66	94.74	98.63	95.86
5	94.58	93.29	97.33	96.19
6	93.38	93.67	95.43	96.35
7	91.88	92.79	98.11	94.83
8	94.76	94.28	97.21	96.92
9	93.71	95.77	96.78	97.03
10	94.43	94.36	97.02	96.74
11	95.01	94.96	96.75	98.05
12	91.33	93.47	95.87	96.74
13	93.71	95.61	97.23	95.39
14	95.44	94.35	98.39	96.54
15	96.36	95.81	96.56	97.06
16	95.27	92.57	97.76	97.88
17	93.19	93.55	96.83	96.43
18	92.32	95.18	97.73	95.78
19	94.79	94.83	97.39	94.35
20	95.32	93.44	98.46	96.79
Average	94.17	94.38	97.21	96.48

**Table 4:** Accuracy and macro-F1 of gait recognition using DPF-LSTM-CNN, DCNN, and CNN-LSTM.

Models	DPF-LSTM-CN	DCNN	CNN-LSTM
Accuracy	97.21%	95.37%	94.57%
Macro-F1	96.48%	94.86%	95.38%

## Data Availability

Data are unavailable due to privacy or ethical restrictions.
